# Tibial lengthening using a retrograde magnetically driven intramedullary lengthening device in 10 patients with preexisting ankle and hindfoot fusion

**DOI:** 10.1080/17453674.2020.1807222

**Published:** 2020-08-24

**Authors:** Bjoern Vogt, Robert Roedl, Georg Gosheger, Gregor Toporowski, Andrea Laufer, Christoph Theil, Jan Niklas Broeking, Adrien Frommer

**Affiliations:** a Children’s Orthopedics, Deformity Reconstruction and Foot Surgery, University Hospital of Muenster; b General Orthopedics and Tumor Orthopedics, University Hospital of Muenster, Germany

## Abstract

Background and purpose — Motorized intramedullary lengthening nails (ILNs) have been developed as an alternative to external fixators for long bone lengthening. The antegrade approach represents the standard method for tibial ILN insertion. In patients with preexisting ankle and hindfoot fusion a retrograde approach provides an alternative technique that has not been evaluated so far. We report the outcome of this method in 10 patients.

Patients and methods — This retrospective study included 10 patients (mean age 18 years [13–25]) with preexisting ankle and hindfoot fusion who underwent tibial lengthening with a retrograde ILN (PRECICE). The mean leg length discrepancy (LLD) was 58 mm (36–80). The underlying conditions were congenital (n = 9) and post tumor resection (n = 1). The main outcome measures were: ILN reliability, distraction achieved, distraction index (DIX), time to bone healing, consolidation index (CIX), complications, and functional results.

Results — All patients achieved the goal of lengthening (mean 48 mm [26–80]). Average DIX was 0.6 mm/day (0.5–0.7) and mean CIX was 44 days/cm (26–60). Delayed consolidation occurred in 2 patients and healed after ILN dynamization or nail exchange with grafting. Toe contractures in 2 other patients were resolved with physiotherapy or tenotomy. Until last follow-up (mean 18 months [12–30]) no true complications were encountered, knee motion remained unaffected, and full osseous consolidation occurred in all patients.

Interpretation — In patients with LLD and preexisting ankle and hindfoot fusion distal tibial lengthening using a retrograde ILN is a reliable alternative to the standard approach with equivalent bone healing potential and low complication rates leaving the knee unaffected.

Fully implantable intramedullary lengthening nails (ILNs) with mechanical (Guichet and Casar 1997, Cole et al. 2001) and motorized (Baumgart et al. 1997, Schiedel et al. 2014) drive systems have been developed as an alternative to external fixators for bone lengthening (Mahboubian et al. 2012, Black et al. 2015, Laubscher et al. 2016). Recently, magnetically driven ILNs in particular have become increasingly popular (Kirane et al. 2014, Wagner et al. 2017) and in contrast to external fixation provide an equally safe and more comfortable option for limb lengthening and deformity correction (Szymczuk et al. 2019, Horn et al. 2019). Antegrade or retrograde femoral and antegrade tibial lengthening with the PRECICE limb lengthening system (NuVasive, San Diego, CA, USA) has been assessed by several studies (Kirane et al. 2014, Schiedel et al. 2014, Shabtai et al. 2014, Tiefenboeck et al. 2016, Wiebking et al. 2016, Fragomen and Rozbruch 2017, Wagner et al. 2017, Iobst et al. 2018, Cosic and Edwards 2020, Nasto et al. 2020).

In tibial lengthening the antegrade approach represents the standard method for ILN implantation (Fragomen and Rozbruch 2017). In patients with preexisting ankle and hindfoot fusion a retrograde approach provides an alternative technique for tibial nail insertion. Approach-associated affections of the knee joint like anterior knee pain (Rothberg et al. 2019) and—in immature patients—damage to the proximal tibial growth plate (Wagner et al. 2017, Frommer et al. 2018) can be avoided. Despite these potential advantages, the use of a retrograde tibial nailing approach and distal tibial osteotomy in patients with preexisting ankle and hindfoot fusion has not been evaluated so far.

## Patients and methods

### Patients and indications

**Figure 1.**
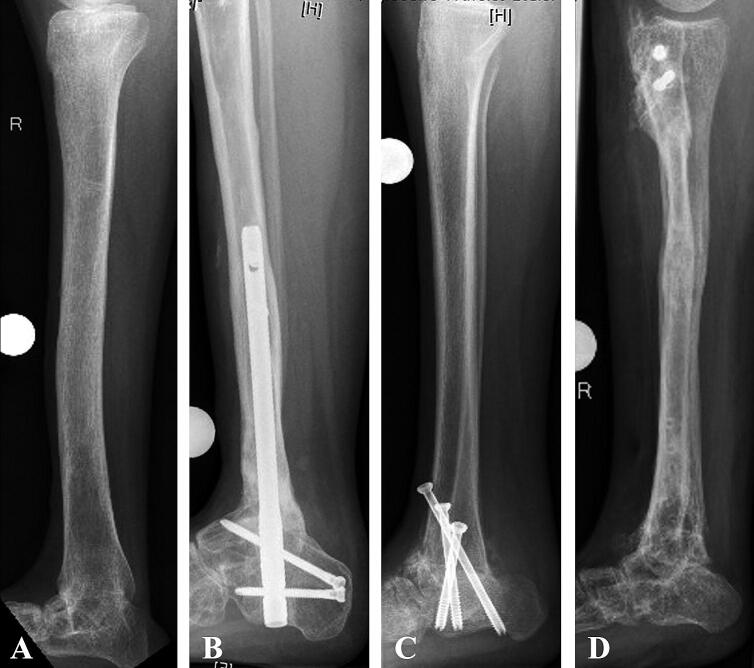
Different etiologies of ankle and hindfoot fusion in patients who were considered for tibial lengthening with retrograde ILN: A—congenital ankylosis (Patient No. 9), B—post tumor resection, subsequent segment transport, and docking with hindfoot nail (Patient No. 1), C—corrected deformity with screw arthrodesis in congenital clubfoot (Patient No. 2), D –fusion achieved with prior external fixation in tibial hemimelia (Patient No. 8).

We performed a retrospective analysis of patients treated with retrogradely implanted magnetically driven ILNs for tibial lengthening between 2015 and 2019. 10 patients (6 females) with preexisting ankle and hindfoot fusion (right = 8) were treated for correction of leg-length discrepancy (LLD). The underlying conditions were congenital (n = 9) and post tumor resection (n = 1) (Table 1). Ankle and hindfoot fusion was established by arthrodesing operations either precedingly in 8 patients or 1-stage with the ILN insertion in 1 individual. A congenital ankylosis was present in 1 patient (Figure 1). The mean preoperative LLD was 58 mm (36–80). 3 patients presented additional persistent angular deformities of the tibia (Nos 4, 6, and 9). The mean period between prior arthrodesis and intramedullary lengthening procedure was 23 months (5–62) (Table 1).

**Table 1.** Patient data—preoperative parameters

Patient no.	Sex	Side	Underlying etiology	No. of previous surgeries ^a^	Previous lengthening	Previous deformity correction	Ankle and hindfoot fusion	Months between fusion and ILN	Preop. LLD (mm)	Persistent deformity
1	F	Left	Tumor resection (osteosarcoma)	> 5	1, femur (ILN)	1, tibia (nail)	Prior 2-stage, (nail)	16	44	No
2	F	Right	Congenital clubfoot	1	No	No	Prior 2-stage, (screws)	6	36	No
3	F	Left	Pes calcaneus in caudal regression syndrome	1	No	No	Prior 2-stage, (nail)	12	38	No
4	F	Right	Fibular hemimelia	> 5	1, femur (ILN) 1, tibia (exFix)	2, femur (1 GG, 1 ILN)	Ankylosis	N/a	55	Distal valgus and flexion
5	F	Right	Fibular hemimelia	> 5	1, tibia (exFix)	2, tibia (1 exFix, 1 nail)	Prior 2-stage, (nail)	16	60	No
6	M	Right	Fibular hemimelia	> 5	2, tibia (exFix)	3, tibia (2 exFix, 1 GG)	1-stage (ILN)	N/a	72	Distal flexion
7	F	Right	Nail-patella syndrome	1	No	No	Prior 2-stage, (screws)	62	60	No

8	M	Right	Tibial hemimelia	1	1, tibia (exFix)	1, tibia (exFix)	Prior 2-stage, (exFix)	60	55	No
9	M	Right	Fibular hemimelia	> 5	1, femur (exFix) 2, tibia (exFix)	3, femur (1 exFix, 2 GG) 3, tibia (2 exFix, 1 GG)	Prior 2-stage, (exFix)	5	75	Mid-shaft valgus
10	M	Right	Tibial hemimelia	> 5	1, tibia (exFix)	3, tibia (1 exFix, 1 nail, 1 GG)	Prior 2-stage, (nail)	10	80	No

**^a^**in affected leg

LLD—leg length discrepancy, ILN—intramedullary lengthening nail, exFix—external fixator, GG—growth guidance, N/a—not applicable.

The mean age at the date of ILN implantation was 18 years (13–25). The planned distraction distance averaged 49 mm (26–80) and thus was 9.0 mm (0–22) shorter than the LLD (Table 2).

**Table 2.** Patient data—perioperative parameters

Patient no.	Age at surgery (years)	ILN diameter/ /stroke (mm)	Concomitant operations	Osteotomy level (mm)	Deformity correction	Surgery time cut to suture (min)	Fluor- oscopy time (min)	Intra- operative injuries	Planned distraction (mm)
1	21.3	8.5/50	Implant removal from prior surgeries	82.9 (meta-diaphyseal)	No	127	3.0	No	36
2	16.9	10.7/50	Implant removal from prior surgeries	84.8 (diaphyseal)	No	156	0.9	No	26
3	18.0	8.5/50	Implant removal from prior surgeries	62.8 (meta-diaphyseal)	No	105	2.5	No	33
4	17.0	10.7/50	No	107.6 (diaphyseal)	Single osteotomy, distal varization and extension	126	3.0	No	40
5	16.1	8.5/80	Implant removal from prior surgeries, tibiotalar bone grafting, screw osteosynthesis	66.9 (meta-diaphyseal)	No	141	1.9	No	55
6	13.3	10.7/50	No	52.3 (meta-diaphyseal)	Single osteotomy, distal extension	83	1.4	No	50
7	20.9	8.5/50	No	80.6 (meta-diaphyseal)	No	89	2.7	No	50
8	24.9	8.5/50	No	52.1 (meta-diaphyseal)	No	128	4.7	No	50
9	17.7	10.7/80	Plate stabilization of second osteotomy	70.4 (meta-diaphyseal)	Second osteotomy, mid-shaft varization	167	5.1	No	65
10	14.7	10.7/80	Epiphysiodesis of proximal fibula	112.2 (diaphyseal)	No	91	1.1	No	80

### Implants and surgical technique

**Figure 3.**
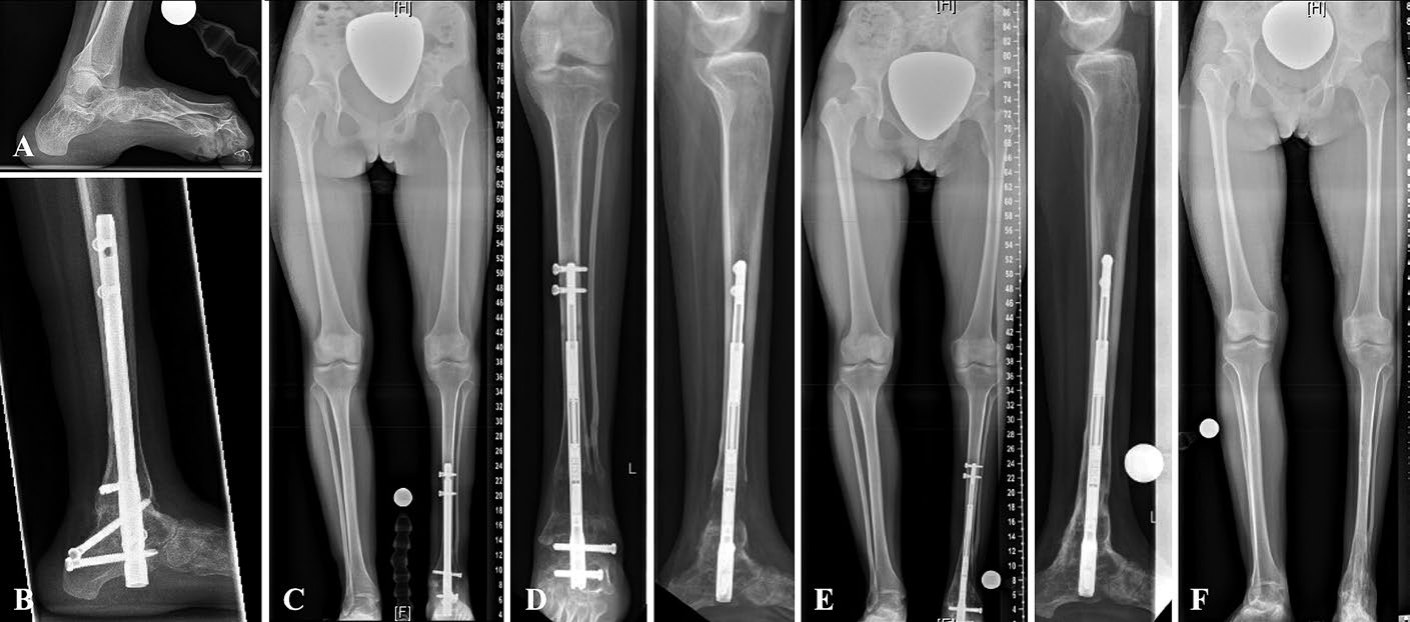
A—Patient No. 3 with pes calcaneus and leg shortening due to caudal regression syndrome. B—Prior to lengthening the foot deformity was corrected by ankle and subtalar arthrodesis using a retrograde nail. C—After ankle and hindfoot fusion the residual LLD measured 38 mm. D—Tibial distraction osteogenesis of 31 mm using a retrograde magnetically driven ILN. E—Postoperative long standing radiograph after full consolidation. F—Postoperative long standing radiograph after ILN removal 26 months postoperatively showing intended residual LLD of 7 mm.

**Figure 4.**
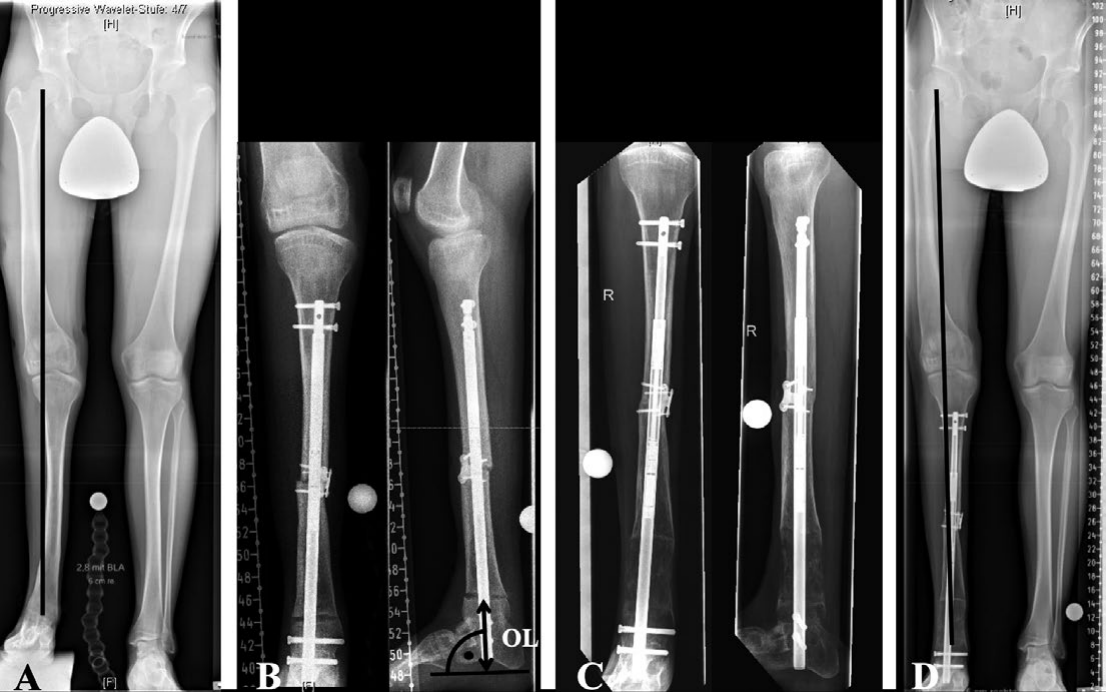
Patient No. 9 with additional osteotomy for angular correction. A—Preoperative radiographs showing LLD of 75 mm and mid-shaft tibial valgus malalignment. B—A second osteotomy was performed on the apex of the deformity for angular correction in addition to a distal corticotomy for callus distraction. The osteotomy level (OL) is measured as the distance from the tibial osteotomy site (drawn as a line parallel to the nail) to a perpendicular line aligned tangentially with the most plantar aspect of the calcaneus on the lateral radiograph (70.4 mm, meta-diaphyseal). The mid-shaft osteotomy was bridged by the ILN and additionally fixed using a locking plate to prevent proximal distraction. C—Full consolidation of the mid-shaft osteotomy and beginning callus consolidation after distraction of 65 mm. D—Postoperative radiograph showing a residual LLD of 11 mm and correction of the tibial valgus malalignment.

The second generation PRECICE (P2) limb-lengthening system (NuVasive, San Diego, CA, USA) was used in all individuals (Table 2, Figures 3–4, for Figures 2 and 5, see Supplementary data). The patients were placed in a supine position on a radiolucent surgical table. All nails were implanted retrogradely according to the preoperative planning (Figure 2, see Supplementary data). The corticotomy was performed on the distal tibia with a multiple drill hole technique and subsequent chiseling. In patients with distal malalignment (Nos 4 and 6) a single osteotomy was carried out on the apex deformity for realignment and callus distraction. In the patient with mid-shaft valgus deformity (No. 9) a second osteotomy was executed on the apex of the deformity for angular correction in addition to a distal corticotomy for callus distraction. The mid-shaft osteotomy was bridged by the ILN and additionally fixed using a 4-hole 3.5 mm locking plate (VariAx, Stryker, Kalamazoo, MI, USA) to prevent proximal distraction (Figure 4). If present, the fibula was osteotomized at the border from the proximal to the distal third (n = 7). Adequate function of the implanted ILN was fluoroscopically proven by intraoperative distraction of 1 mm. In 6 patients 7 concomitant operations were performed (Table 2).

### Pre- and postoperative clinical and radiographic evaluation

The patients underwent clinical and radiographic evaluations preoperatively and periodically after surgery until final follow-up.

The radiographs were taken using the Centricity PACS calibrated digital radiology system (GE Healthcare, Chalfont St Giles, UK). All radiographic measurements were performed with the TraumaCad (Brainlab, Munich, Germany) post-processing software. Anteroposterior (AP) long standing radiographs were obtained preoperatively and at last follow-up in order to assess LLD and coronal alignment of the lower extremity (Paley 2002) (Figures 3 and 4). In immature patients (Nos 6 and 10) final LLD was predicted using the multiplier method (Paley et al. 2000).

### Postoperative lengthening and follow-up protocol

Distraction commenced after a latency period of 10 days with an initial distraction speed of 0.66 mm/day in all patients. The follow-up protocol involved clinical and radiographic examinations every 2 weeks during the lengthening period and every 6 weeks during consolidation. The patients were allowed 20 kg partial weight-bearing during distraction and after osseous consolidation full weight-bearing was permitted. All patients were treated with physiotherapy at least once a week during the lengthening period. The average postoperative follow-up was 18 months (12–30).

### Outcome parameters

#### Perioperative parameters

Implant type, surgery time including concomitant operations (cut–suture), fluoroscopy time, and amount of intraoperative blood loss were acquired from the surgery protocol (Table 2).

#### Functional outcome

The presence of postoperative pain was assessed. Postoperative functional results were evaluated using the AOFAS ankle–hindfoot scale.

#### Limb-lengthening parameters

The osteotomy level (OL) was measured on lateral radiographs and categorized by the corresponding anatomical bone segment (Figure 4B). The amount of distraction achieved was calculated subtracting the length of the initially exposed male portion of the nail from the length of the extended male portion at the end of distraction. Accuracy, precision, and reliability were calculated as previously described (Schiedel et al. 2014, Wagner et al. 2017). The distraction index (DIX) was determined by dividing the achieved length (mm) by the duration of lengthening (days). The consolidation index (CIX) was calculated by dividing the time from surgery until full weight-bearing was possible (days) by the distraction length (cm). Delayed osseous consolidation (non-union) was defined as absent bone healing 6 months after the end of distraction.

#### Limb alignment parameters

Post-surgical bone results were documented according to Chappell et al. (2019). This includes the ankle and hindfoot alignment grading as proposed by Katsenis et al. (2005) and the tibial alignment grading as proposed by Schoenleber and Hutson (2015).

#### Difficulties

In accordance with Paley (1990) difficulties during the lengthening procedure were subclassified into problems, obstacles, and true complications.

### Ethics, funding and potential conflicts of interest

The study was approved by the ethical committee of the university of Muenster on July 1, 2019 (registration number: 2019-368-f-S) and was fully financed by the research funds of the University Hospital of Muenster, Germany. BV and RR are paid consultants of NuVasive (San Diego, USA) and have received remuneration during the study period outside the submitted work. The other authors have no conflict of interest.

## Results

### Surgical procedure and perioperative parameters

No intraoperative injuries were recorded. The mean surgery and fluoroscopy time including concomitant operations was 121 minutes (83–167) and 2.6 minutes (0.9–5.1), respectively, with an average documented blood loss of 258 mL (80–500). No perioperative blood transfusion was necessary. Corticotomies were conducted 7 times on the metadiaphyseal and 3 times on the diaphyseal region with a mean OL of 77 mm (52–112) (Table 2).

### Limb-lengthening parameters

All 10 patients successfully completed the lengthening procedures. The average achieved distraction measured 48 mm (26–80). The mean difference between the planned and the actual distraction was –1.1 mm (–2 to 0). The accuracy of distraction was 97.9% (SD 2.2) and the precision was 97.8%. The mean distraction period was 82 days (50–128) and the mean consolidation period was 6.6 months (5.1–8.9). The mean DIX was 0.6 mm/day (0.5–0.7) and CIX 1.5 days/cm (0.9–2.0). All lengthening procedures were completed with the ILN remaining in situ until the end of distraction showing excellent reliability (100%). At the time of manuscript submission (April 30, 2020) 7 ILNs were explanted after a mean of 16 months (7–35). Residual postoperative LLD measured 8.9 mm (0–22). According to Chappell et al. (2019) the parameter “LLD” was excellent in 8 patients and good in 2 cases (Nos 4 and 6) (Table 3).

**Table 3.** Patient data—postoperative parameters

A	B	C	D	E	F	G	H	I	J	K	L	M	N	0	P	Q
1	35	–1	9	59	N/A ^a^	0.63	N/A ^a^	Delayed wound healing, acceleration of distraction rate	Non-union	No	E	E	83 (G)	Shoe finishing	Done	30.1
2	26	0	10	51	5.1	0.51	1.9	Deceleration of distraction rate		No	E	E	84 (G)	Orthopedic shoes	Done	15.9
3	31	–2	7	50	6.2	0.62	2.0	Toe flexion contracture		No	E	E	86 (G)	Orthopedic shoes	Done	26.3
4	38	–2	17	57	7.1	0.67	1.9	Toe flexion contracture, deceleration of distraction rate		No	E	E	86 (G)	None	Done	21.6
5	53	–2	7	85	N/A ^a^	0.62	N/A ^a^	Delayed wound Healing	Non-union	No	E	E	81 (G)	Orthopedic Shoes	Done	19.5
6	50	0	22	93	5.1	0.54	1.0	Delayed wound healing		No	G	E	79 (F)	Orthosis	Scheduled	14.9
7	49	–1	11	78	6.5	0.65	1.3	Pain	Slight ILN bending	No	E	E	86 (G)	Orthopedic shoes	Done	12.3
8	50	0	5	95	8.9	0.53	1.8	Pain, deceleration of distraction rate	Slight ILN bending **^b^**	No	E	E	77 (F)	Orthosis	Done	12.3
9	64	–1	11	121	6.7	0.53	1.0		Slight ILN bending	No	E	E	86 (G)	Orthosis	Scheduled	13.0
10	80	0	0	128	7.0	0.63	0.9	Pain, acceleration of distraction rate		No	E	E	81 (G)	Orthosis	Scheduled	12.8

**^a^**Non-union

APatient no.

BAchieved distraction (mm)

CDifference achieved – planed distraction (mm)

DFinal leg length discrepancy (mm)

EDistraction period (days)

FConsolidation period (months)

GDistraction index (mm/d)

HConsolidation index (months/cm)

IProblems

JObstacles

ILN—intramedullary lengthening nail

**^b^**preventive nail exchange

KTrue complications

LKatsenis-Hindfoot-Alignment score

E­—Excellent

G—Good

MSchoenleber-Tibia-Alignment score

E­—Excellent

NAOFAS-Score

G—Good

F—Fair

OOrthopaedic assist devices

PILN removal

QFollow-up (months)

N/A—Not applicable

### Limb alignment parameters

The Hindfoot-Alignment Score according to Katsenis et al. (2005) was excellent in 9 patients and good in 1 case. The Tibia-Alignment Score according to Schoenleber and Hutson (2015) was excellent in all patients. According to Chappell et al. (2019) the subscales “deformity hindfoot” and “deformity tibia” correspond to the aforementioned scores.

### Difficulties

#### Problems

3 patients complained continuously of moderate pain under distraction alleviated by an oral non-steroidal antiphlogistic.

Delayed wound healing was successfully treated nonoperatively in 3 patients. Flexion contractures of the toes due to relative tendon shortening occurred in 2 patients and were resolved either by physiotherapy (No. 3) or tenotomy and arthrodesis during ILN removal (No. 4).

In half of the patients an adjustment of the distraction rate was necessary due to either the risk of premature consolidation (temporary acceleration = 2) or insufficient callus formation and toe flexion contractures (temporary deceleration = 3) (Table 3).

#### Obstacles

No additional surgeries were necessary under distraction. Non-union led to a prolonged course of treatment in 2 patients. Full osseous consolidation was achieved by proximal dynamization of the nail (No. 1) or nail exchange combined with autologous bone grafting (No. 5) (Figure 5, see Supplementary data).

Another nail exchange was performed preventively in a patient (No. 8) who did not abide by the weight-bearing restrictions. Premature callus consolidation was not observed. No implant-associated complications were documented. Minor ILN bending (< 5°) was found in 3 patients. No deep infections were recorded.

#### True complications

No true complications were observed. No intraoperative injuries were recorded. All difficulties during limb lengthening were resolved before the end of treatment. No additional surgery was necessary after consolidation. No lengthening procedure failed to achieve the planned distraction by more than 1 cm.

### Functional outcome

Range of motion of the knee was not affected by the lengthening procedure in any patient. According to the AOFAS ankle–hindfoot score grading categories (maximum score 100) 8 patients reached a good and 2 patients a fair result. The average AOFAS ankle–hindfoot score measured 83 (77–86). Due to the preexisting ankle and hindfoot fusion the maximum score in our cohort is 86 instead of 100 (Rochman et al. 2008).

At the time of manuscript submission all patients were pain free and able to ambulate under full weight-bearing load without walking aids. All except 1 patient (No. 4) relied on an orthosis or shoe customizing for ambulation pre- and postoperatively (Table 3).

## Discussion

This is the first reported series of tibial lengthening through retrogradely implanted ILNs in patients with preexisting ankle and hindfoot fusion.

The basic idea regarding the preference for a retrograde approach for tibial ILN implantation in contrast to the standard antegrade insertion technique is to avoid an entry-related effect on the knee joint. Antegrade tibial nail implantation always requires a knee arthrotomy (Fragomen and Rozbruch 2017) and bears the risk of joint effusion or hemarthrosis associated with pain and restricted ROM. Some patients develop persisting anterior knee pain and may become unable to kneel (Rothberg et al. 2019). Due to the retrograde ILN implantation inherently no approach-related effects on the knee joint were observed in our patients. On the other hand, plantar nail entry carries the risk of approach-related problems such as painful soft tissue irritation or delayed wound healing (Mosca et al. 2020). 3 of our patients had delayed plantar wound healing. No cases of deep infections or neurovascular injury occurred.

In patients undergoing proximal tibial lengthening procedures using antegrade ILNs persistent knee joint contractures have been observed in up to 40% (Shabtai et al. 2014, Tiefenboeck et al. 2016, Wiebking et al. 2016, Nasto et al. 2020), whereas persistent ROM restrictions of the ankle joint were encountered in up to 25% of patients (Kirane et al. 2014, Shabtai et al. 2014, Tiefenboeck et al. 2016, Wiebking et al. 2016, Cosic and Edwards 2020, Nasto et al. 2020). In our series all patients had a fused ankle and hindfoot, and consequential development of equinus contracture or other ankle or hindfoot deformity did not present a problem. No patient developed temporary or permanent ROM restrictions of the knee joint. Toe-associated problems such as flexor tendon contractures were encountered in 2 patients (Nos 3 and 5). However, toe clawing resolved by flexor tendon release has also been described following proximal tibial lengthening using antegrade ILNs (Kirane et al. 2014, Tiefenboeck et al. 2016).

Patients with an unstable knee due to congenital deficiencies are exposed to a risk of knee dislocation (Shabtai et al. 2014, Black et al. 2015, Szymczuk et al. 2019). Despite 6 of our 10 patients having a congenital deficiency, no knee dislocation was observed. We believe that distal tibial distraction via retrogradely inserted ILN might especially be beneficial for patients with an unstable knee and preexisting ankle and hindfoot fusion, i.e., due to congenital deficiencies.

To prevent physeal damage in immature patients antegrade ILN implantation is considered to be contraindicated and consequently lengthening is usually carried out using external fixators (Wagner et al. 2017, Frommer et al. 2018). In patients with preexisting ankle and hindfoot fusion and concomitant closure of the distal tibial growth plate, the retrograde approach allows the use of a tibial ILN and eliminates the need for external fixators. The distraction goal should be defined on the basis of the predicted LLD at skeletal maturity when conducting leg lengthening in immature patients with a closed distal tibial physis (Frommer et al. 2018). In patients with fused ankle and hindfoot the goal of the treatment should not be exact LLD equalization. An intentional under-correction to a residual LLD of 0.5–1.0 cm is advisable to permit adequate “swing-through” during gait (McCoy et al. 2012). Achieving this goal can become challenging in skeletally immature patients.

Tibial ILN insertion through an antegrade approach is technically demanding (Fragomen and Rozbruch 2017) and susceptible to the development of iatrogenic deformities (Lee et al. 2017). Usually blocking screws are necessary to prevent flexion and coronal (especially valgus) deformities (Kirane et al. 2014, Fragomen and Rozbruch 2017, Lee et al. 2017). On the other hand, the same techniques facilitate deformity corrections close to the knee joint (Fragomen and Rozbruch 2017). Using a retrograde ILN with a distal tibial osteotomy, knee-associated deformities can hardly be addressed. In our patients only diaphyseal or distal residual deformities of the tibia were simultaneously realigned during ILN implantation. Although most of the patients had congenital conditions that are usually associated with coronal tibial deformities close to the knee joint (especially valgus), the tibial alignment grading by Schoenleber and Hutson (2015) showed excellent results in all patients as most of the patients had previously undergone deformity reconstructions either by antegrade nail or external fixator.

Using a retrograde ILN the corticotomy for distraction osteogenesis is located in the distal tibia. Several studies investigated the results of tibial lengthening with ankle arthrodesis using external fixation. Most authors carried out proximal tibial distractions of 40–55 mm and found an external fixation index of 54–76 days/cm. Non-union of the regenerate was found in 4–25% of the patients (Katsenis et al. 2005, Rochman et al. 2008, Tellisi et al. 2008, Fragomen et al. 2012). Only a few studies evaluated the results of ankle and hindfoot reconstruction and concurrent tibial lengthening through a distal corticotomy. The described distraction index was 0.55 mm/day and the external fixation index was 70–144 days/cm. Non-union occurred in 0–10% of patients. In accordance with our findings the authors demonstrated successful tibial lengthening via a distal corticotomy but did not recommend this approach for distractions of more than 3–4 cm (Sakurakichi et al. 2003, Chappell et al. 2019). Based on our observations, sufficient callus regenerate and bone healing in the distal tibia can also be achieved after longer distraction distances. These findings might indicate that the osseous healing potential of proximal or distal tibial distraction osteogenesis is equivalent.

Independent of concomitant ankle and hindfoot pathologies various authors have analyzed proximal tibial lengthening procedures with antegrade magnetically driven ILNs (Kirane et al. 2014, Schiedel et al. 2014, Shabtai et al. 2014, Tiefenboeck et al. 2016, Wiebking et al. 2016, Wagner et al. 2017, Cosic and Edwards 2020, Nasto et al. 2020). The achieved distraction was 26–45 mm (Schiedel et al. 2014, Wiebking et al. 2016, Cosic and Edwards 2020). The DIX was reported as 0.48–0.84 mm/day (Tiefenboeck et al. 2016, Wiebking et al. 2016, Wagner et al. 2017, Nasto et al. 2020) and the CIX amounted to 0.5–3.3 months/cm (Shabtai et al. 2014, Tiefenboeck et al. 2016, Horn et al. 2019, Cosic and Edwards 2020, Nasto et al. 2020). Non-union was encountered in 11–75% (Kirane et al. 2014, Shabtai et al. 2014, Tiefenboeck et al. 2016, Wiebking et al. 2016, Wagner et al. 2017, Cosic and Edwards 2020, Nasto et al. 2020). These findings are comparable to our results for distal tibial distraction osteogenesis using a retrograde ILN with non-union in one-fifth of patients and a CIX of 1.5 months/cm, indicating at least equivalent bone healing potential.

Slight bending (< 5°) of the ILN occurred in 3 patients. However, this radiographic finding was not associated with malfunction of the nail, diminished bone healing, or secondary malalignment of the tibia. In all of these cases the distraction distance was close to or greater than 50 mm and the smallest nail diameter of 8.5 mm was chosen in 2 of these patients. Thus, greater lengthening distances with increasing leverage and smaller nail diameters with reduced strength of the device might be risk factors for ILN bending. Whenever an 8.5 mm ILN was implanted, this was due to the limited anatomy of the hindfoot or tibia, taking into account the necessary amount of over-reaming.

This study has limitations due to its retrospective, observational character and the small number of patients treated with only 1 type of ILN. In order to identify potential risk factors for treatment failure prospective groupwise and comparative study designs are needed, i.e, comparison with antegrade tibial ILN. The results of the study do not provide any comparison with the potential benefit of tibial lengthening with external fixators or other types of ILNs. There is a selection bias towards congenital etiologies, which is explained by the specification of our department.

In summary, distal tibial lengthening via a retrograde ILN is a reliable alternative to proximal tibial distraction and antegrade ILN insertion for correction of LLD in patients with preexisting ankle and hindfoot fusion, showing equivalent bone healing potential and low complication rates, and avoiding approach-related effects on the knee. Monitoring of callus consolidation and critical assessment of potential soft tissue complications, especially of the toes under distraction, are mandatory.

## Supplementary Material

Supplemental Material

## Supplementary data

Figures 2 and 5 are available as supplementary data in the online version of this article, http://dx.doi.org/10.1080/17453674.2020.1807222

## References

[CIT0001] Baumgart R, Betz A, Schweiberer L. A fully implantable motorized intramedullary nail for limb lengthening and bone transport. Clin Orthop Relat Res 1997; (343): 135–43. 9345218

[CIT0002] Black S R, Kwon M S, Cherkashin A M, Samchukov M L, Birch J G, Jo C H. Lengthening in congenital femoral deficiency: a comparison of circular external fixation and a motorized intramedullary nail. J Bone Joint Surg Am 2015; 97(17): 1432–40. 26333739 10.2106/JBJS.N.00932 PMC7535106

[CIT0003] Chappell T M, Ebert C C, McCann K M, Hutchinson B L, Rodriguez-Collazo E. Distal tibial distraction osteogenesis: an alternative approach to addressing limb length discrepancy with concurrent hindfoot and ankle reconstruction. J Orthop Surg Res 2019; 14(1): 244. 31362774 10.1186/s13018-019-1264-0 PMC6668173

[CIT0004] Cole J D, Justin D, Kasparis T, DeVlught D, Knobloch C. The intramedullary skeletal kinetic distractor (ISKD): first clinical results of a new intramedullary nail for lengthening of the femur and tibia. Injury 2001; 32(Suppl 4): SD129–39. 11812486 10.1016/s0020-1383(01)00116-4

[CIT0005] Cosic F, Edwards E. PRECICE intramedullary nail in the treatment of adult leg length discrepancy. Injury 2020. 10.1016/j.injury.2020.03.004 32164952

[CIT0006] Fragomen A T, Rozbruch S R. Lengthening and deformity correction about the knee using a magnetic internal lengthening nail. SICOT J 2017; 3: 25. 28322717 10.1051/sicotj/2017014 PMC5360097

[CIT0007] Fragomen A T, Borst E, Schachter L, Lyman S, Rozbruch S R. Complex ankle arthrodesis using the Ilizarov method yields high rate of fusion. Clin Orthop Relat Res 2012; 470(10): 2864–73. 22777590 10.1007/s11999-012-2470-9 PMC3441986

[CIT0008] Frommer A, Rodl R, Gosheger G, Vogt B. [Application of motorized intramedullary lengthening nails in skeletally immature patients: indications and limitations]. Unfallchirurg 2018; 121(11): 860–7. 30203390 10.1007/s00113-018-0541-4

[CIT0009] Guichet J M, Casar R S. Mechanical characterization of a totally intramedullary gradual elongation nail. Clin Orthop Relat Res 1997; (337): 281–90. 10.1097/00003086-199704000-00032 9137201

[CIT0010] Horn J, Hvid I, Huhnstock S, Breen A B, Steen H. Limb lengthening and deformity correction with externally controlled motorized intramedullary nails: evaluation of 50 consecutive lengthenings. Acta Orthop 2019; 90(1): 81–7. 30371122 10.1080/17453674.2018.1534321 PMC6366464

[CIT0011] Iobst C A, Rozbruch S R, Nelson S, Fragomen A. Simultaneous acute femoral deformity correction and gradual limb lengthening using a retrograde femoral nail: technique and clinical results. J Am Acad Orthop Surg 2018; 26(7): 241–50. 29494464 10.5435/JAAOS-D-16-00573

[CIT0012] Katsenis D, Bhave A, Paley D, Herzenberg J E. Treatment of malunion and nonunion at the site of an ankle fusion with the Ilizarov apparatus. J Bone Joint Surg Am 2005; 87(2): 302–9. 15687151 10.2106/JBJS.C.01421

[CIT0013] Kirane Y M, Fragomen A T, Rozbruch S R. Precision of the PRECICE internal bone lengthening nail. Clin Orthop Relat Res 2014; 472(12): 3869–78. 24682741 10.1007/s11999-014-3575-0 PMC4397804

[CIT0014] Laubscher M, Mitchell C, Timms A, Goodier D, Calder P. Outcomes following femoral lengthening: an initial comparison of the Precice intramedullary lengthening nail and the LRS external fixator monorail system. Bone Joint J 2016; 98-B(10): 1382–8. 27694593 10.1302/0301-620X.98B10.36643

[CIT0015] Lee D H, Kim S, Lee J W, Park H, Kim T Y, Kim H W. A comparison of the device-related complications of intramedullary lengthening nails using a new classification system. Biomed Res Int 2017; 2017: 8032510. 29130046 10.1155/2017/8032510 PMC5654310

[CIT0016] Mahboubian S, Seah M, Fragomen A T, Rozbruch S R. Femoral lengthening with lengthening over a nail has fewer complications than intramedullary skeletal kinetic distraction. Clin Orthop Relat Res 2012; 470(4): 1221–31. 22143986 10.1007/s11999-011-2204-4 PMC3293955

[CIT0017] McCoy T H, Goldman V, Fragomen A T, Rozbruch S R. Circular external fixator-assisted ankle arthrodesis following failed total ankle arthroplasty. Foot Ankle Int 2012; 33(11): 947–55. 23131440 10.3113/FAI.2012.0947

[CIT0018] Mosca M, Caravelli S, Fuiano M, Massimi S, Censoni D, Grassi A, Vocale E, Ceccarelli F, Zaffagnini S. Tibiotalocalcaneal arthrodesis through retrograde nailing for the treatment of juxtaarticular distal tibia aseptic non-unions: a retrospective study at a minimum follow-up of 4 years. Injury 2020; 51(6): 1377–81. 32327237 10.1016/j.injury.2020.03.039

[CIT0019] Nasto L A, Coppa V, Riganti S, Ruzzini L, Manfrini M, Campanacci L, Palmacci O, Boero S. Clinical results and complication rates of lower limb lengthening in paediatric patients using the PRECICE 2 intramedullary magnetic nail: a multicentre study. J Pediatr Orthop B 2020. [Online ahead of print] 10.1097/BPB.0000000000000651 31904740

[CIT0020] Paley D. Problems, obstacles, and complications of limb lengthening by the Ilizarov technique. Clin Orthop Relat Res 1990; (250): 81–104. 2403498

[CIT0021] Paley D. Principles of deformity correction. Berlin/New York: Springer; 2002.

[CIT0022] Paley D, Bhave A, Herzenberg J E, Bowen J R. Multiplier method for predicting limb-length discrepancy. J Bone Joint Surg Am 2000; 82(10): 1432–46. 11057472 10.2106/00004623-200010000-00010

[CIT0023] Rochman R, Jackson Hutson J, Alade O. Tibiocalcaneal arthrodesis using the Ilizarov technique in the presence of bone loss and infection of the talus. Foot Ankle Int 2008; 29(10): 1001–8. 18851816 10.3113/FAI.2008.1001

[CIT0024] Rothberg D L, Stuart A R, Presson A P, Haller J M, Higgins T F, Kubiak E N. A comparison of the open semi-extended parapatellar versus standard entry tibial nailing techniques and knee pain: a randomized controlled trial. J Orthop Trauma 2019; 33(1): 31–6. 30211787 10.1097/BOT.0000000000001309

[CIT0025] Sakurakichi K, Tsuchiya H, Uehara K, Kabata T, Yamashiro T, Tomita K. Ankle arthrodesis combined with tibial lengthening using the Ilizarov apparatus. J Orthop Sci 2003; 8(1): 20–5. 12560881 10.1007/s007760300003

[CIT0026] Schiedel F M, Vogt B, Tretow H L, Schuhknecht B, Gosheger G, Horter M J, Rodl R. How precise is the PRECICE compared to the ISKD in intramedullary limb lengthening? Reliability and safety in 26 procedures. Acta Orthop 2014; 85(3): 293–8. 24758320 10.3109/17453674.2014.913955 PMC4062798

[CIT0027] Schoenleber S J, Hutson J J, Jr. Treatment of hypertrophic distal tibia nonunion and early malunion with callus distraction. Foot Ankle Int 2015; 36(4): 400–7. 25358806 10.1177/1071100714558509

[CIT0028] Shabtai L, Specht S C, Standard S C, Herzenberg J E. Internal lengthening device for congenital femoral deficiency and fibular hemimelia. Clin Orthop Relat Res 2014; 472(12): 3860–8. 24664194 10.1007/s11999-014-3572-3 PMC4397748

[CIT0029] Szymczuk V L, Hammouda A I, Gesheff M G, Standard S C, Herzenberg J E. Lengthening with monolateral external fixation versus magnetically motorized intramedullary nail in congenital femoral deficiency. J Pediatr Orthop 2019; 39(9): 458–65. 31503231 10.1097/BPO.0000000000001047

[CIT0030] Tellisi N, Fragomen A T, Ilizarov S, Rozbruch S R. Limb salvage reconstruction of the ankle with fusion and simultaneous tibial lengthening using the Ilizarov/Taylor spatial frame. HSS J 2008; 4(1): 32–42. 18751860 10.1007/s11420-007-9073-0 PMC2504274

[CIT0031] Tiefenboeck T M, Zak L, Bukaty A, Wozasek G E. Pitfalls in automatic limb lengthening: first results with an intramedullary lengthening device. Orthop Traumatol Surg Res 2016; 102(7): 851–5. 27527249 10.1016/j.otsr.2016.07.004

[CIT0032] Wagner P, Burghardt R D, Green S A, Specht S C, Standard S C, Herzenberg J E. PRECICE((R)) magnetically-driven, telescopic, intramedullary lengthening nail: pre-clinical testing and first 30 patients. SICOT J 2017; 3: 19. 29785927 10.1051/sicotj/2016048 PMC5962966

[CIT0033] Wiebking U, Liodakis E, Kenawey M, Krettek C. Limb lengthening using the PRECICE(TM) nail system: complications and results. Arch Trauma Res 2016; 5(4): e36273. 28144605 10.5812/atr.36273 PMC5253187

