# Supplementary material for: Tibial lengthening using a retrograde magnetically driven intramedullary lengthening device in 10 patients with preexisting ankle and hindfoot fusion
Source: Acta Orthop. 2020 Aug 24;91(6):761–9. doi: 10.1080/17453674.2020.1807222 (PMC8023964; doi:10.1080/17453674.2020.1807222)
Supplement: Supplemental Material [file IORT_A_1807222_SM9035.pdf]

Supplementary data

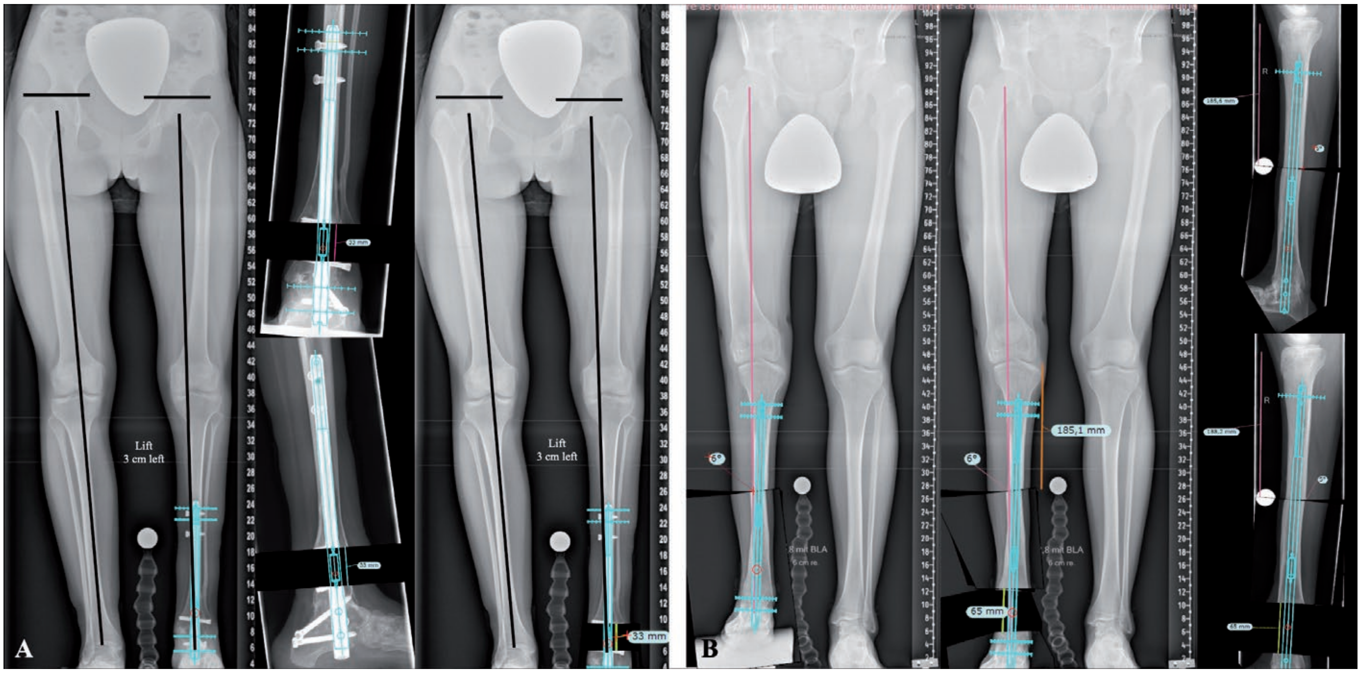

Figure 2. Preoperative planning: A—(Patient No. 3, LLD = 38 mm). Due to symmetric genu varum a concomitant acute coronal plane correction was not considered to be necessary. To preserve the knee joint a retrograde approach through the pre-existing ankle and hindfoot fusion was chosen. An ILN with 8.5 mm diameter, 190 mm initial length, and 50 mm stroke was planned. The choice of nail diameter is limited by the width of the medullary canal. The planned distraction distance of 33 mm (intentional under-correction to a residual LLD of 5 mm) was simulated. B—(Patient No. 9, LLD = 75 mm). Due to the mid-shaft tibial valgus malalignment a second osteotomy was planned on the apex of the deformity for varization of 6° in addition to a distal osteotomy for distraction of 65 mm. In the lateral plane the osteotomy compensated a slight antecurvation of 5° on the same level approximately 185 mm distal to the knee joint. To avoid strain on the unstable knee joint a retrograde ILN insertion with distal tibial distraction was chosen. Diameter (10.7 mm), initial length (335 mm), and stroke (80 mm) of the ILN was planned.

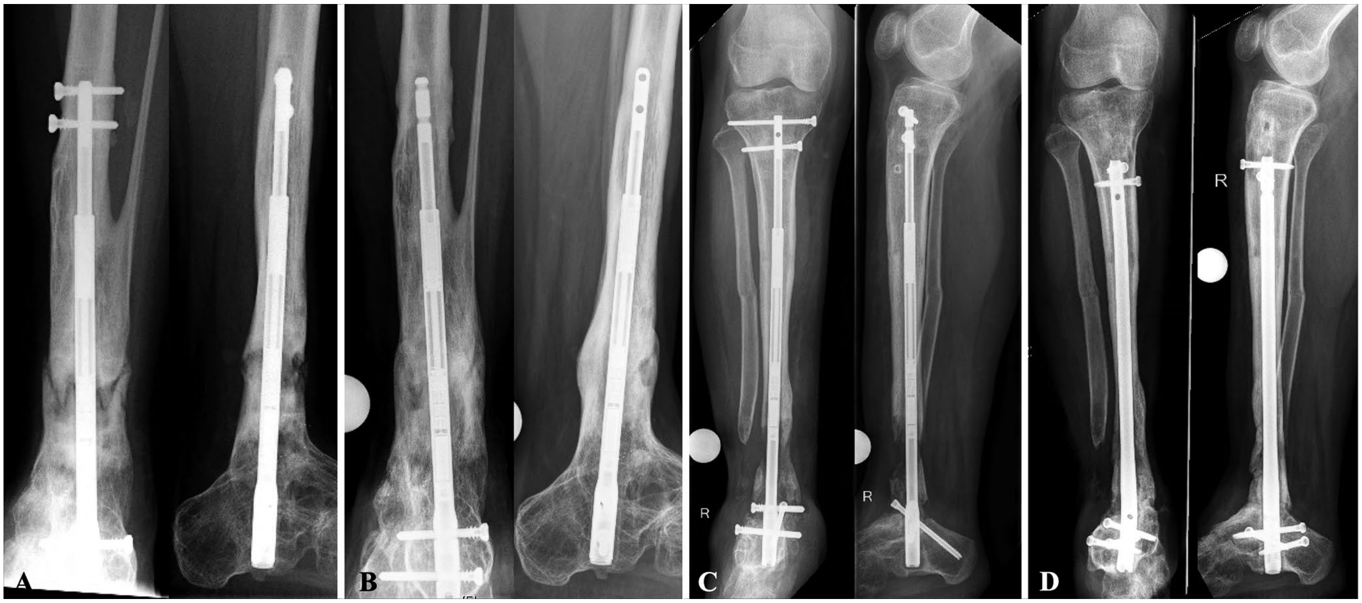

Figure 5. Patients with non-union (A–B Patient No. 1, C–D Patient No. 5). A—Non-union after ILN-controlled distal tibial lengthening. B—Full consolidation after dynamization of the ILN by removal of the proximal locking screws. C—Non-union after ILN-controlled distal tibial lengthening. D—Full consolidation after autologous bone grafting and exchange of the ILN to a trauma nail.
